# Cutaneous Tuberculosis With Multisystem Involvement in an Immunocompetent Refugee

**DOI:** 10.7759/cureus.95623

**Published:** 2025-10-28

**Authors:** Nabor S Mireles, Natalie Garcia, Omid Jalali

**Affiliations:** 1 Medicine, Baylor College of Medicine, Houston, USA; 2 Dermatology, Baylor College of Medicine, Houston, USA

**Keywords:** cutaneous tuberculosis, mycobacterium tuberculosis, tuberculosis, tuberculosis chancre, ulcerative tuberculosis

## Abstract

Cutaneous tuberculosis (CTB) is an uncommon skin infection that can mimic other dermatologic conditions and delay recognition. Herein, we present a case of TB chancre, a form of CTB caused by exogenous inoculation. A 19-year-old immunocompetent male refugee from Sudan presented with a five-month history of non-healing wounds on his right shoulder and three weeks of bilateral lower extremity weakness. Diagnostic examination revealed the presence of a spinal epidural abscess and lung consolidation, with culture of the spinal abscess positive for *Mycobacterium tuberculosis* (MTB). Skin biopsy of the shoulder lesion revealed granulomatous dermatitis, with subsequent culture confirming MTB from the cutaneous wound at approximately six weeks and from sputum at eight weeks. During admission, the patient was treated with standard RIPE (rifampin, isoniazid, pyrazinamide, and ethambutol) therapy, resulting in improvement of wound healing and stabilization of neurologic symptoms. This case emphasizes the diagnostic challenges of CTB due to its atypical and non-specific presentation and highlights the importance of considering CTB in the differential diagnosis for chronic, non-healing wounds in patients from endemic areas or with relevant exposure history.

## Introduction

Cutaneous tuberculosis (CTB) is due to infection of the skin by *Mycobacterium tuberculosis* (MTB), an acid-fast bacillus. In the 1980s and 1990s, there was an increase in the incidence of tuberculosis (TB), and therefore, an increase in cases of CTB. This surge is attributed to the HIV epidemic, increased use of immunosuppressive drugs, emergence of resistant strains of TB, migration of people around the world, and a decrease in TB control efforts [1].

Although TB is not particularly virulent, with only 5-10% of infections leading to clinical disease in immunocompetent people, it is estimated that one-quarter of the world’s population is infected with TB. In 2023, 10.8 million people worldwide contracted TB [2]. In 2023, there were 9,615 cases of TB reported in the United States, which was a 16% increase from 2022, and the highest number of cases reported since 2013 [3]. An estimated 85% of TB cases in the US are due to reactivation of latent TB rather than recent transmission. TB is also the leading cause of death worldwide [2]. 

With this increasing incidence of TB, an increasing number of cases of CTB are expected. CTB is relatively uncommon and only accounts for 1-2% of the extracutaneous manifestations of TB [4]. CTB has a wide variety of clinical presentations, including but not limited to tuberculous chancre, TB verrucosa cutis, scrofuloderma, orificial tuberculosis, lupus vulgaris, miliary tuberculosis, and tuberculous gumma [5]. Herein, we present the case of an immunocompetent male patient who was found to have CTB presenting as a non-healing wound.

## Case presentation

A 19-year-old immunocompetent male refugee from Sudan with a past medical history of Strongyloidiasis treated with ivermectin presented to the emergency department with three weeks of bilateral lower leg weakness and unsteady gait. He also had a history of non-healing wounds on his right shoulder for five months. The first wound appeared after he dropped a book on his shoulder and developed a bruise. The second wound appeared spontaneously one month prior to presentation. Previously, he had presented to a dermatology clinic and was prescribed silver sulfadiazine cream, which did not improve wound healing.

He was admitted for workup of bilateral lower extremity weakness, initially expected to be transverse myelitis. Spinal MRI revealed a T1/T2 epidural abscess, requiring emergent surgical intervention for spinal cord decompression with neurosurgery. A CT chest also revealed a left lower lobe consolidation and necrotic mediastinal and perihilar lymphadenopathy. Given immigration status and medical presentation, there was high suspicion for infection with MTB.

While inpatient, he underwent extensive infectious workup (Table 1).

**Table 1:** Microbiologic and histopathologic results with corresponding timing relative to admission Includes QuantiFERON-TB Gold, spinal and cutaneous specimens, and sputum cultures, with final results and time to positivity. H&E, hematoxylin and eosin; AFB, acid-fast bacilli; H&E, hematoxylin and eosin; PAS, periodic acid–Schiff

Specimen / Test	Day 2	2 Weeks	6 Weeks	8 Weeks	Reference range
QuantiFERON-TB Gold	Positive				Negative
Spinal epidural abscess culture		Positive for *Mycobacterium tuberculosis*			No growth
Skin biopsy H&E (right shoulder)	Granulomatous dermatitis with ulceration				Not applicable
Skin biopsy Fite stain	Negative				Negative
Skin biopsy PAS stain	Negative				Negative
Skin biopsy Gram stain	Negative				Negative
Skin biopsy AFB culture			Positive for *Mycobacterium tuberculosis*		No growth
Sputum AFB cultures (six specimens)				Positive (one specimen)	No growth

**Figure 1:**
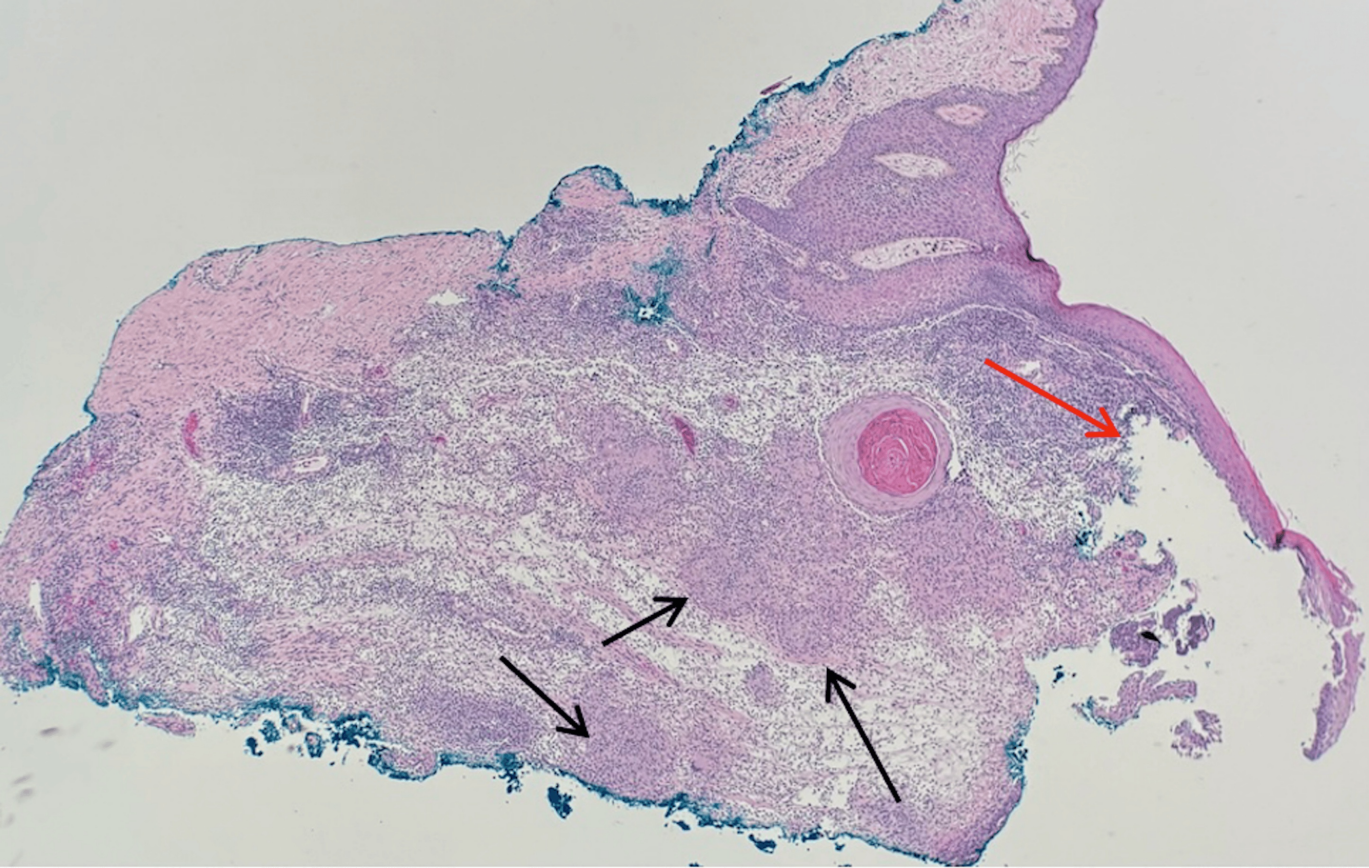
Permanent section, hematoxylin and eosin stain (2x) Shows an ulcerated epidermis (red arrow) overlying multiple dermal necrotizing granulomas (black arrows), supporting a necrotizing granulomatous dermatitis compatible with mycobacterial infection in this clinical context.

**Figure 2:**
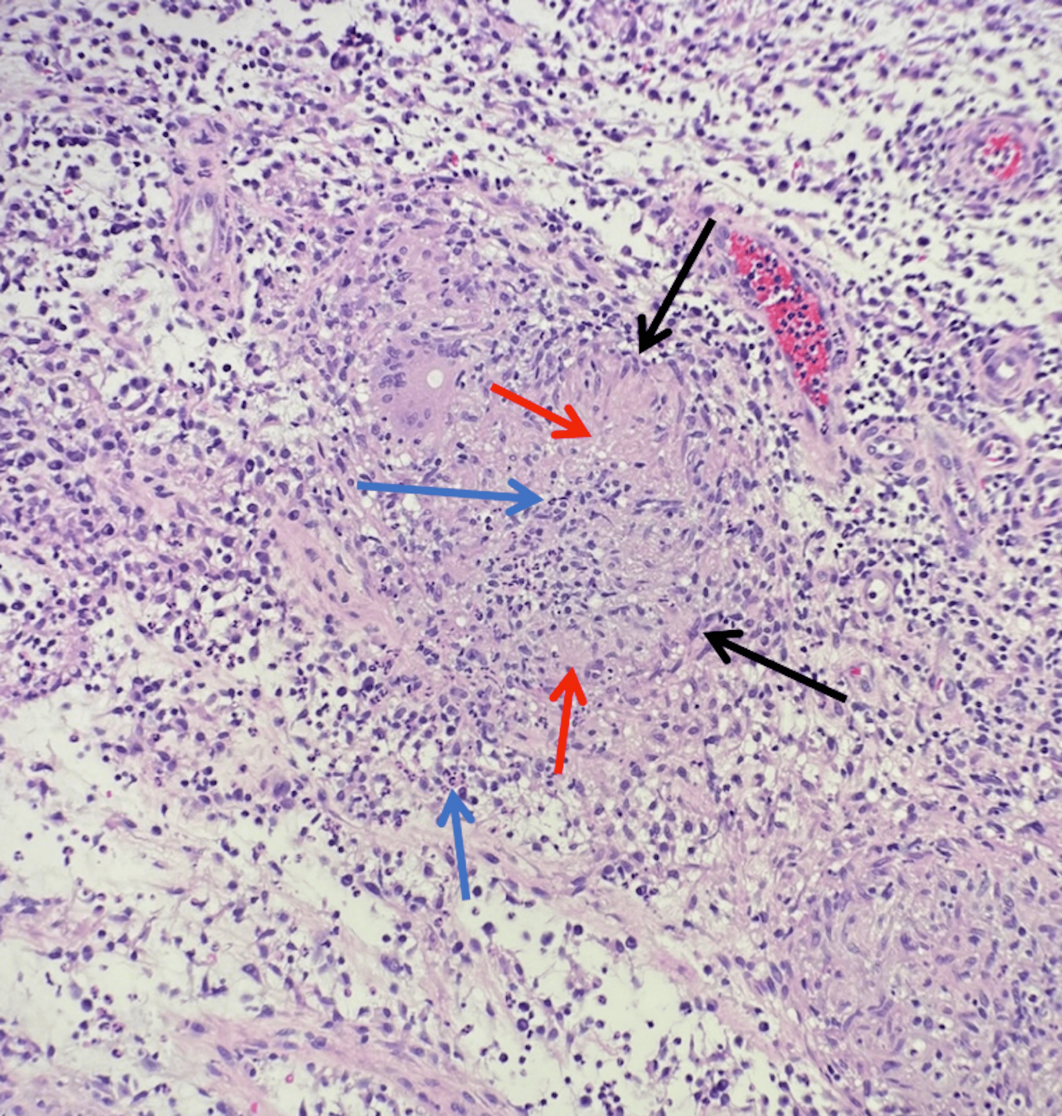
Permanent section, hematoxylin and eosin stain (20x) Demonstrates a suppurative granuloma with central necrosis (red arrows) bordered by epithelioid histiocytes (black arrows) and neutrophils (blue arrows), favoring an infectious etiology.

**Figure 3:**
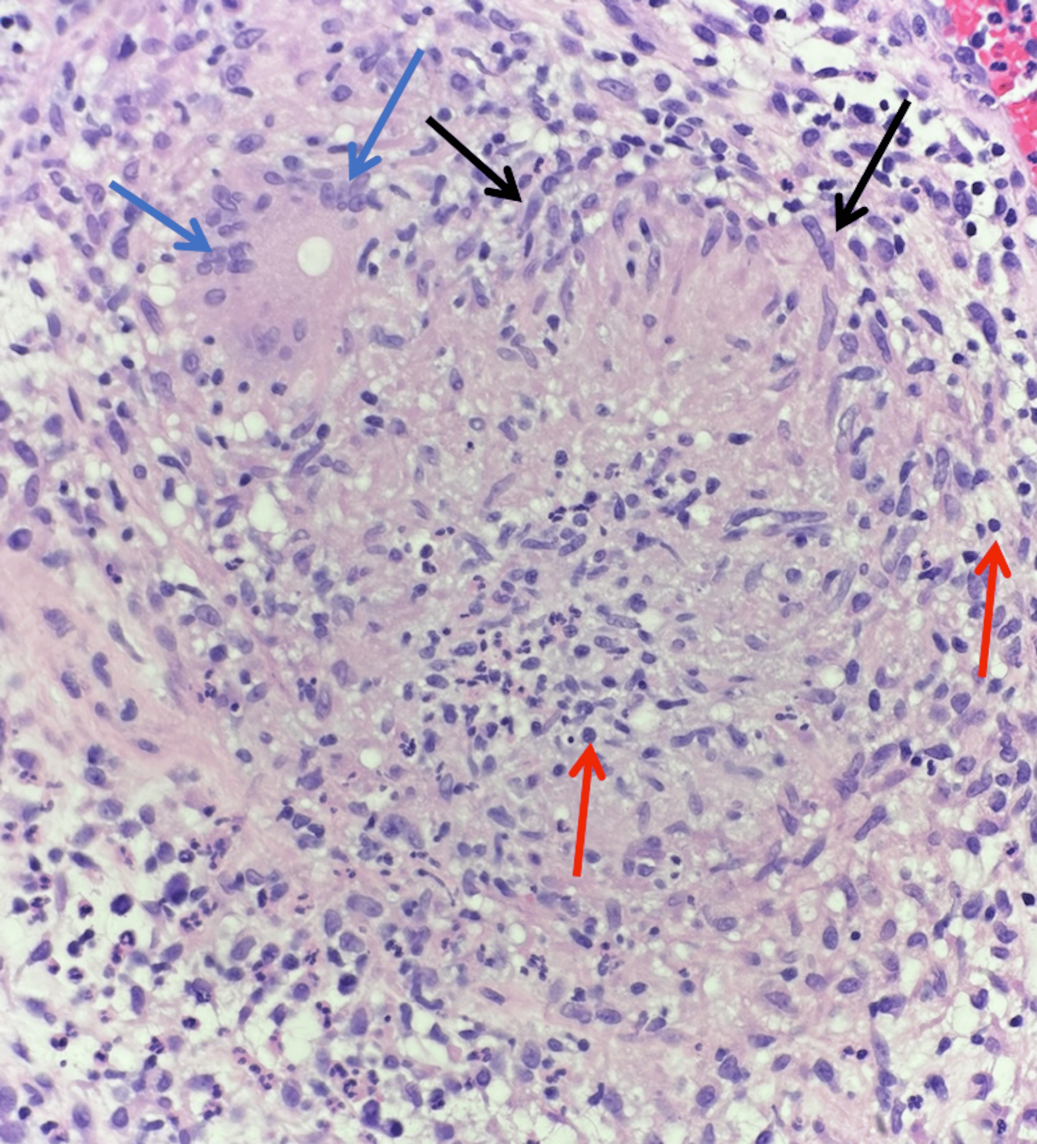
Permanent section, hematoxylin and eosin stain (40x) Shows a well-circumscribed dermal granuloma composed of closely-packed epithelioid histiocytes (black arrows), surrounded by lymphocytes (red arrows) and scattered multinucleated giant cells (blue arrows) aligning with cutaneous tuberculosis (CTB) in the setting of culture positivity.

Initial lab work revealed a positive QuantiFERON-TB Gold (QIAGEN, Germantown, MD, USA) at which point RIPE therapy was started with isoniazid, rifampin, pyrazinamide, ethambutol, and pyridoxine. While on RIPE therapy, his shoulder wounds began to heal. Cultures from his spinal epidural abscess were the first cultures to be obtained and returned positive for *Mycobacterium tuberculosis* about two weeks post-admission. On the same day as the spinal epidural abscess cultures were taken, dermatology performed two 4-mm punch biopsies on the initial wound of his right shoulder. One tissue sample was submitted for hematoxylin and eosin (H&E) staining and another for infectious workup. Biopsy initially revealed granulomatous dermatitis with ulceration (Figures 1-3). 

**Figure 4:**
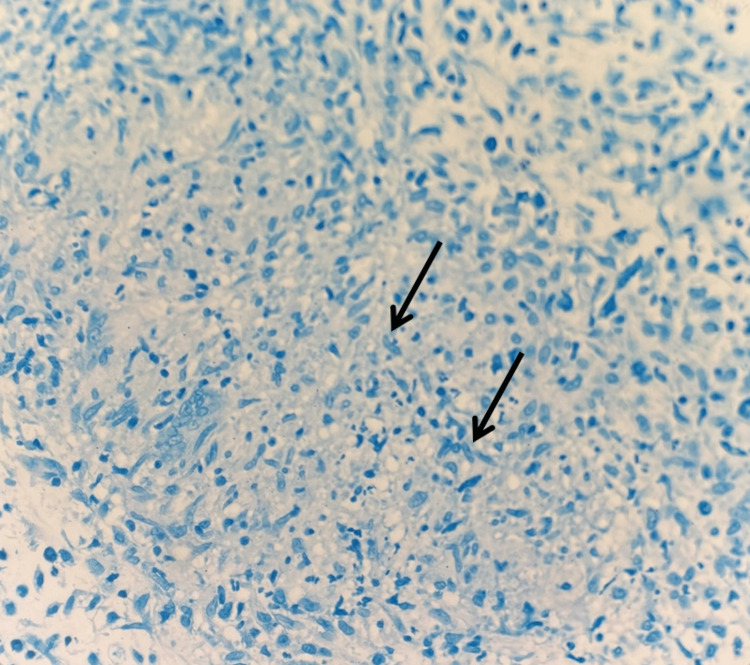
Permanent section, Fite stain (40x) Shows granulomatous dermis (black arrows) without demonstrable acid-fast bacilli. As cutaneous tuberculosis can be paucibacillary, this does not rule out mycobacterial infection.

**Figure 5:**
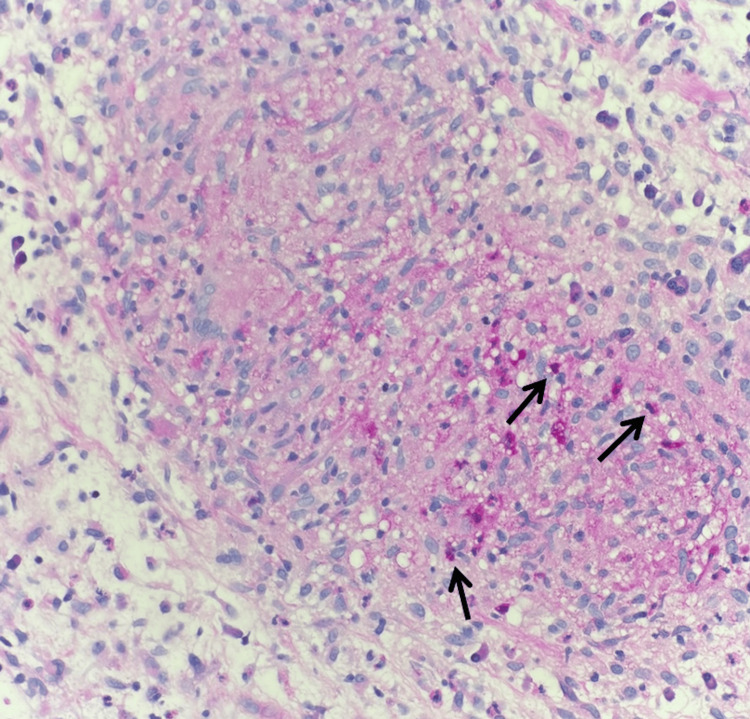
Permanent section, periodic acid–Schiff with diastase stain (20x) This was negative for fungal organisms with scattered neutrophils (black arrows), arguing against a fungal etiology for the granulomatous process.

**Figure 6:**
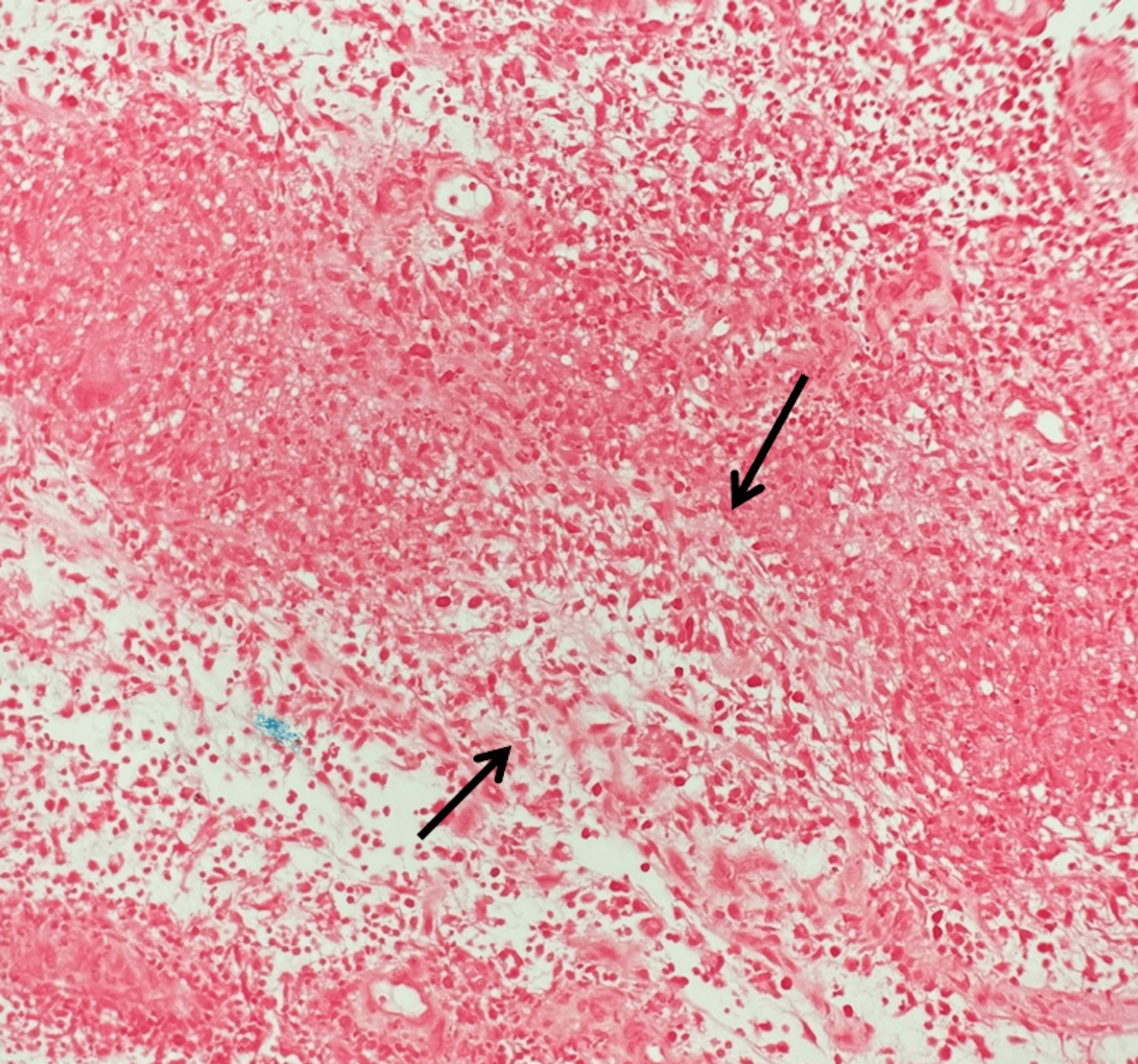
Permanent section, Gram stain (20x) This was negative for bacterial organisms with necrotizing granulomatous tissue indicated (black arrows), supporting a mycobacterial process given the histology and subsequent culture results.

Fite, periodic acid-Schiff (PAS), and Gram stains were negative for microorganisms (Figures 4-6).

About six weeks after obtaining the skin biopsy on the right shoulder, the acid-fast bacilli culture returned positive for MTB, resulting in the diagnosis of CTB. Six sputum acid-fast bacilli cultures were collected, and all except one returned negative for infection. The single positive culture resulted approximately eight weeks after admission, by which time he was discharged on RIPE therapy with plans for close multidisciplinary follow-up with infectious disease, neurosurgery, dermatology, ophthalmology, and primary care.

## Discussion

TB is a chronic granulomatous infection caused by MTB with the potential to impact any organ, most commonly the lungs [4]. CTB is uncommon, representing approximately 1-2% of the extra-pulmonary TB cases, which themselves account for about 8-24% of all TB cases [4,6]. The atypical and variable presentations of CTB frequently mimic other skin conditions, contributing to diagnostic delays [4]. Considering the disease burden, with approximately a quarter of the global population estimated to have been infected, awareness of CTB is crucial, especially in patients with risk factors and exposure history [2].

In 2023, TB regained its distinction as the leading cause of death worldwide from a single infectious agent, surpassing COVID-19 [2]. The World Health Organization reported 10.8 million new TB cases globally in 2023, with five countries (India, Indonesia, China, the Philippines, and Pakistan) accounting for 56% of cases. In contrast, the United States reported 9,615 new TB cases in 2023, an increase of 16% from the previous year, with 76% of cases occurring in non-U.S.-based individuals, and 85% attributed to latent infection [3]. Sudan, where our patient is from, is among the countries where TB is a major health concern, with an estimated 29,000 reported cases in 2019 [7].

The pathogenesis of CTB depends on multiple factors, including load and virulence of the infecting pathogen, the route of infection, prior sensitization, and the host’s immune status [8]. Infection can occur through exogenous inoculation, endogenous spread from an internal source, and hematogenous and lymphatic dissemination [4,8]. The route of infection often influences the clinical variant of CTB that the patient develops. The main variants of CTB include, but are not limited to, TB chancre, TB verrucosa cutis, scrofuloderma, orificial TB, lupus vulgaris, miliary TB, and TB gumma [5].

Only 5-10% of immunocompetent individuals exposed to MTB will progress to develop clinically significant disease [4]. Genetic factors have also been linked to disease onset. Mutations in the interferon-gamma receptor and IL-12 receptor genes have been attributed to an increased risk of TB vulnerability due to an impaired immune response [4]. Comorbid conditions, particularly HIV and immunosuppressive therapies, significantly increase the risk of TB reactivation and dissemination through the depletion of CD4+ T-cells and interferon-gamma production [4,8,9].

However, our patient was immunocompetent, HIV negative, and had no history of immunosuppressive medication use and yet had multi-organ dissemination of TB. Given his non-healing ulceration at the site of a minor injury that occurred months prior to the onset of his neurological symptoms, as well as no history of prior sensitization, including no previous Bacillus Calmette-Guerin (BCG) vaccination, TB chancre is the most likely variant in our patient. TB chancre is exceedingly rare, accounting for less than 1% of all CTB cases [4]. This variant occurs through exogenous infection at the site of primary inoculation in individuals without prior immunity and is characterized by painless ulcers with undermined edges [4]. Histologically, the lesion appears granulomatous with necrotic areas and numerous acid-fast bacilli (AFB) [10]. However, after three to six weeks, the number of AFBs may decrease, which may explain why none were visible with Fite, PAS, or gram staining but were confirmed weeks later via culture [10]. If left untreated, TB chancre has the potential to progress to scrofuloderma or facilitate hematogenous spread, leading to systemic involvement [11]. Thus, timely diagnosis is crucial. However, it is unclear whether the multisystem involvement stemmed from dissemination of the cutaneous lesion or represented parallel manifestations of primary TB infection.

Currently, the diagnosis of CTB requires a high index of clinical suspicion and confirmation through one or more of the following: histologic demonstration of AFB, tissue culture confirmation of AFB, positive tuberculin skin test (TST), or evidence of systemic TB [12]. However, this is often challenging due to its rarity and nonspecific features. In our patient, the differential diagnosis was broad and included TB among other infectious etiologies due to the atypical lesion. Although there was suspicion of TB involvement while awaiting QuantiFERON-TB Gold assay results, the non-specific constellation of symptoms prompted initiation of broad-spectrum antibiotics empirically before initiating TB therapy upon receiving the positive assay result, highlighting the difficulty in prompt confirmation of CTB diagnosis. Other contributors known to create diagnostic delay include the prolonged culture time for MTB (typically three to six weeks, though occasionally longer), overgrowth of commensal skin flora, low sensitivity of cultures due to variable paucity of MTB in skin lesions, and non-specific histological findings [10,13,14]. In these cases, polymerase chain reaction (PCR) testing had been advocated as a rapid and accurate means of diagnosing CTB, with proven success over direct microscopy and comparable results with culture, though limitations exist [8,10].

Treatment options for CTB are limited and typically follow standard RIPE therapy consisting of a two-month intensive phase with rifampin, isoniazid, pyrazinamide, and ethambutol, followed by a continuation phase with isoniazid and rifampin for approximately four months [12,15]. Most variants respond well to this treatment and carry a good prognosis [10]. Occasionally, surgical intervention may be required [6]. No topical therapy for CTB has been identified [8]. Our patient responded well to RIPE therapy with adequate progress in wound care throughout his admission and was discharged to follow up with a TB clinic while continuing his RIPE regimen.

Dermatologists and other clinicians should maintain a high index of suspicion for CTB when evaluating chronic or non-healing lesions, especially when examining patients from endemic areas or with relevant exposure history. In such scenarios, a TB test could also assist with the diagnosis.

## Conclusions

This rare case of an immunocompetent 19-year-old male patient with CTB presenting as TB chancre highlights the importance of considering CTB in the differential diagnosis of chronic, non-healing skin lesions, especially in patients from endemic areas or with a history of recent exposure. Although uncommon, CTB can lead to significant morbidity if not adequately treated. Dermatologists and other clinicians should maintain a high index of suspicion for CTB in patients with chronic or non-healing wounds, especially in patients from endemic areas or with relevant exposure histories.
